# Students’ Perceptions of their Dental Curriculum and Education

**DOI:** 10.12669/pjms.38.6.5183

**Published:** 2022

**Authors:** Aljazi H. Aldweesh, Mohammed Aldhubaiban, Alanoud Alqahtani, Iman Emad Almohammad, Fares S. Al-Sehaibany, Sahar F. Albarakati

**Affiliations:** 1 Aljazi Hussain Aldweesh BDS, Msc, PhD. Associate Professor, Division of Orthodontics Department of Pediatric Dentistry and Orthodontics : College of Dentistry, King Saud University, Riyadh, Saudi Arabia; 2 Mohammed Abdulaziz Aldhubaiban BDS, MDS. Assistance Professor, Dept. of Pediatric Dentistry & Orthodontics, College of Dentistry, King Saud University, Riyadh, Saudi Arabia; 3 Alanoud Abdullah Alqahtani, BDS. Endodontic Resident, King Faisal Specialist Hospital Research Center, Riyadh, Saudi Arabia; 4 Iman Emad Almohammad, BDS. Endodontic Resident, Dammam Medical Complex, Dammam, Saudi Arabia; 5 Fares S. Al-Sehaibany Professor, Department of Pediatric Dentistry and Orthodontics, : College of Dentistry, King Saud University, Riyadh, Saudi Arabia; 6 Sahar F Albarakati, BDS, MS, Cert Ortho. Professor, Division of Orthodontics Department of Pediatric Dentistry and Orthodontics, : College of Dentistry, King Saud University, Riyadh, Saudi Arabia

**Keywords:** Dental training, Dental curriculum, Education, Satisfaction

## Abstract

**Objective:**

To evaluate the satisfaction of dental students toward their dental curriculum and education at dental colleges in the central region of Saudi Arabia.

**Methods:**

Two forms of the questionnaire were established, a paper version and an electronic internet-based survey (Google online form). Paper versions were distributed to interns graduated from universities in Riyadh. The electronic versions were used to obtain responses from students at dental colleges and universities outside of Riyadh, such as Prince Sattam bin Abdulaziz University (PSAU) and Majmaah University, by emailing the links to the participants who were dental interns that had graduated in the 2018/2019 academic year.

**Results:**

A total of 388 interns answered the questionnaire. Overall, 48.4% of the interns were highly satisfied with the Operative Department while regarding Orthodontic Department 16.9% were satisfied while 11.2% were highly satisfied. Regarding the non-clinical satisfaction score, the interns were mostly unsatisfied with their research skills (15.5%). Comparing the interns’ satisfaction at different institutions, there was a statistically significant difference in the clinical satisfaction score (P ≤0.01), but there was no difference in the non-clinical satisfaction score (P > 0.05).

**Conclusion:**

Dental students need a greater focus and exposure to research skills during their dental school studies. To improve dental students’ level of clinical satisfaction, it is more important for them to have early exposure to comprehensive clinical training than specialty-based clinical training.

## INTRODUCTION

Most graduate dental students are committed to being a safe general practitioner, applying their knowledge, competence, and skills in the clinical practice of dentistry.^1^ Moreover, they must possess critical thinking and problem-solving skills to evaluate health care issues and have good communication skills and know how to use technologies.^2^ Previous studies have stated that acquiring skills does not just entail transmission of information from the teacher to the learner; it is more advanced process.^3^-^6^

The General Dental Council’s (GDC) guidelines emphasizes that all members of the dental team should develop and maintain their skills and knowledge throughout their career. Clinical, communication, professionalism, management, and leadership are the four domains that are mentioned in that guidelines.^1^ Oliver et al. concluded that most dental students felt that they did not receive enough training on some practical skills in dental schools, such as surgical endodontics (76%), conscious sedation (72%), root surface debridement (71%), fixed orthodontics appliance (68%), porcelain veneers (63%), implants (56%), and posterior composite (53%).^1^ Moreover, the respondents mentioned that they needed to improve most of their skills and the dental curriculum should be updated to address non-clinical subjects, such as business and practice management (21%) and communications skills (10%), and increase the amount of clinical time (8%).^1^ Therefore, dental schools need to continuously modify their curriculum to ensure that they adapt to developments in knowledge, clinical practices, and oral health needs.^7^ The most important tool to evaluate the quality of dental curriculum is monitoring the level of confidence and satisfaction that dental students have in their dental education.^8^ Thus, the present study aimed to evaluate the satisfaction of dental interns toward their dental curriculum and education at colleges and universities in the central region of Saudi Arabia.

## METHODS

This cross-sectional study was carried out after getting the ethical approval by KSUNo.18/0658/IRB October 15, 2018. Two forms of the questionnaire were established, a paper version and an electronic internet-based survey (Google online form). Paper versions were distributed to interns graduated from universities inside Riyadh. The electronic versions were used to obtain responses from dental graduates from dental colleges and universities outside of Riyadh, such as Prince Sattam bin Abdulaziz University (PSAU) and Majmaah University. A pilot study on 30 participants was first carried out to assess its clarity. The participants were asked if the questionnaire questions were easy to fill and doesn’t take time. Results of the pilot study revealed that the questionnaire was easy to understand and participants didn’t face any difficulty filling it up.

The surveys were distributed randomly by emailing the links/distributing questionnaire papers to 500 dental interns that had graduated in the 2018/2019 academic year. The inclusion criteria were males and females’ dental interns who graduated from dental schools and are in their internship year and their dental school was in the central region. The exclusion criteria were students who are still studying in the dental school or who have been finished their internship year or their dental school was outside the central region. The questionnaire was designed to assess their satisfaction with their clinical and non-clinical skills. It aimed to collect information on three areas:

### Demographic data

gender, age, and the participants’ dental school.

• The participants’ opinions of the general clinical skills they acquired during their dental education.
• The participants’ opinions of the non-clinical skills they acquired during their dental education.

In the questionnaire, each question required a response using a five-point scale: 1) highly satisfied, 2) satisfied, 3) neutral, 4) unsatisfied, and 5) highly unsatisfied. Participation in the survey was voluntary, and all the responses were anonymous. The participants signed a consent form before they completed the paper version of the questionnaire. For the online survey, the participants’ consent was obtained by completion and submission of the questionnaire.

At a confidence interval 95% and an alpha of 0.05, the total sample size should be at least 371. Due to the nature of an online survey, only a 70–80% response rate was expected. The survey was distributed to 500 interns. Data were collected and analyzed using statistical package for social science (SPSS) software v.22 (IBM Corp., NY, USA). A one-way analysis of variance (ANOVA) and t-test were used. Statistical significance was set at a P-value of < 0.05.

## RESULTS

The response rate was 77.6%; a total of 388 interns completed the questionnaire. Based on the demographic data analysis, 56% of the participants were female and 44% were male. Of the participants, 33% were interns at Riyadh Elm College, about 29% were interns at King Saud University (KSU), 16% were at interns at Alfarabi College, 9% were from King Saud Bin Abdulaziz for Health Sciences (KSAUH), 6% were from Prince Nora University (PNU), 4% were from Prince Sattam Bin Abdulaziz University (PSAU), and 3% were from Majmaah University. The participants were asked about their satisfaction level with the different departments of their colleges or universities.

The results identified that the majority of the participants (48.1%) were satisfied with the clinic in the Endodontics Department, 26.9% were neutral, 18.1% were highly satisfied, 6.2% were unsatisfied, and 0.8% highly unsatisfied. Most of the interns in our study sample were highly satisfied (48.4%) with the Operative Department, 38.3% were satisfied, 11.1% were neutral, 1.3% were unsatisfied, and 0.8% were highly unsatisfied. Most of the interns (41.9%) were satisfied with the Pediatric Dentistry Clinic, 28.4% were highly satisfied, 23.3% were neutral, 3.6% were unsatisfied, and 2.8% were highly unsatisfied.

**Fig.1:**
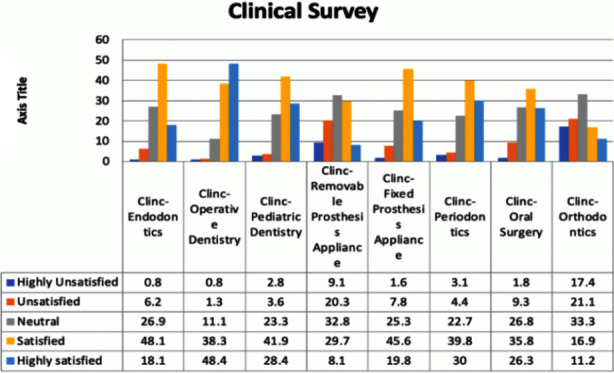
Clinical Survey.

The interns’ opinions about the Clinical-Removable Prosthesis Department were mixed; 32.8% were neutral, 29.7% were satisfied, 20.3% were unsatisfied, 19.8% were highly satisfied, and 9.1% were highly unsatisfied. Regarding the Clinical Fixed Prosthesis Department, 45.6% of the interns were satisfied, 25.3% were neutral, 19.8% were highly unsatisfied, 7.8% were unsatisfied, and 1.6% were unsatisfied. Regarding the Periodontics Clinic, 39.8% of the interns were satisfied, 30% were highly satisfied, 22.7% were neutral, 4.4% were unsatisfied, and 3.1% were highly unsatisfied. Furthermore, 35.8% of the participants were satisfied with the Oral Surgery Department, 26.8% were neutral, 26.3% were highly satisfied, 9.3% were unsatisfied, and 1.8% were highly unsatisfied. Lastly, regarding Orthodontics Clinic, 33.3% of the interns were neutral, 21.1% were unsatisfied, 17.4% were highly unsatisfied, 16.9% were satisfied, 11.2% were highly satisfied (Fig.1).

**Fig.2:**
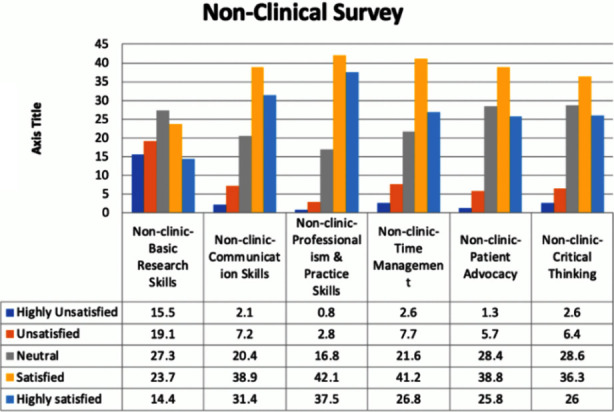
Non-Clinical Survey.

The survey results related to the interns’ satisfaction with their non-clinical skills is presented in Fig-2. The results showed that the majority of the participants (27.3%) were neutral about the basic research skills taught in the colleges and universities, 23.7% were satisfied, 14.4% were highly satisfied, 19.1% were unsatisfied, and 15.5% were highly unsatisfied. Concerning communication skills, 38.9% of the interns were satisfied, 31.4% were highly satisfied, 20.4% were neutral, 7.2% were dissatisfied, and 2.1% were highly unsatisfied. As regards professionalism, most of the participants were satisfied, 37.5% were highly satisfied, 16.8% were neutral, 2.8% were unsatisfied, and 0.8% were highly unsatisfied. Similarly, for and practice skills most of the interns (41.2%) were satisfied, 26.8% were highly satisfied, 21.6% were neutral, 7.7% were unsatisfied, and 2.6% were highly unsatisfied. As regards patient advocacy, 38.8% of the interns were satisfied, 28.4% were neutral, 25.8% were highly satisfied, 5.7% were unsatisfied, and 1.3% were highly unsatisfied. Regarding non-clinic critical thinking skills, 36.3% of the interns were satisfied, 28.6% were neutral, 26% were highly unsatisfied, 6.4% were unsatisfied, and 2.6% were highly unsatisfied. The overall results showed that most of the interns were satisfied with their professionalism and practice skills and time management skills. However, most of them were unsatisfied with their research skills. (Fig.2).

A t-test was also used to determine the overall satisfaction level among the male and female interns. As seen in Table-I, the results of the analysis indicate that there is no difference between females and males in terms of their overall clinical satisfaction level (P = 0.014). The female interns had a higher overall non-clinical satisfaction score than the male interns (P ≤0.01).

**Table I:** Differences between male and female interns in Clinical and non-Clinical satisfaction scores.

Type	Gender	N	Mean	Std. Deviation	T-test P-value
Overall clinic % score	Male	169	64.105	13.583	0.014
	Female	219	67.765	15.190	
Overall, none clinic % score	Male	169	64.128	19.069	0.01
	Female	219	71.957	18.922	

Results presents in Table-II of the ANOVA that was implemented to determine the participants’ overall satisfaction level with the clinical and non-clinical factors relative to the dental colleges and universities. There was a statistically significant difference in the overall clinical satisfaction based on the colleges and universities (P ≤ 0.01). The participants’ overall clinic satisfaction score was highest for PNU (mean score of 76.884), followed by KSAUH, and then Majmaah University. The participants were least satisfied with the clinical performance of KSU. However, there was no statistically significant difference in the overall non-clinical satisfaction level (P > 0.05), although the satisfaction level of the PNU interns is higher in relation to the clinical aspects, followed by KSAUH. Riyadh Elm College was ranked third in terms of the overall non-clinical satisfaction score.

**Table II:** Comparison of interns’ clinical and non-clinical satisfaction scores among different Universities.

Type	College	n	Mean	Std. Deviation	ANOVA p-value	95% Confidence Interval for Mean			Multiple Comparison Test					
	
						Lower Bound	Upper Bound	KSU	Riyadh Elm	KSAUH	PNU	Al Farabi College	Majmaah	PSAU
Overall clinic % score	KSU	113	60.634	13.007	0.01	58.209	63.058	1						
	Riyadh Elm	127	66.915	13.664		64.515	69.314	0.063	1					
	KSAUH	35	73.140	14.759		68.070	78.210	0.002	0.489	1				
	PNU	25	76.884	13.435		71.338	82.430	0.000	0.103	0.984	1			
	Al Farabi College	61	66.107	17.064		61.736	70.477	0.416	1.000	0.466	0.106	1		
	Majmaah	12	69.308	12.423		61.415	77.202	0.652	0.999	0.995	0.881	0.997	1	
	PSAU	15	65.213	8.257		60.641	69.786	0.964	1.000	0.759	0.367	1.000	0.997	1

Overall, none clinic % score	KSU	113	62.240	18.011	9.000	58.883	65.597	1						
	Riyadh Elm	127	72.802	16.589		69.889	75.716	0.004	1					
	KSAUH	35	76.189	19.403		69.523	82.854	0.019	0.988	1				
	PNU	25	80.840	15.768		74.331	87.349	0.002	0.678	0.988	1			
	Al Farabi College	61	61.070	23.565		55.035	67.106	1.000	0.011	0.022	0.003	1		
	Majmaah	12	70.142	19.374		57.832	82.451	0.919	1.000	0.987	0.840	0.875	1	
	PSAU	15	70.833	10.798		64.854	76.813	0.822	1.000	0.989	0.836	0.758	1	1

^*^KSU: King Saud University; PSAU: Prince Sattam Bin Abdulaziz University; PNU: Prince Nora University; KSAUH: King Saud Bin Abdulaziz for Health Science.

## DISCUSSION

The Bachelor of Dentistry (B.D.S) degree program normally comprises of four years of formal education while in some countries, it can span over a period of six years.^9^,^10^ Dental education is usually divided into two parts i.e. pre-clinical and clinical years.^10^,^11^ In the pre-clinical years, students are mostly studying basic science subjects didactically and their practical work is restricted to dental laboratories where they practice on the phantom head/extracted teeth.^10^,^12^ In the clinical years, students have a bit of didactic part but most of their work is based in the clinical environment, where they work on real patients under the supervision of specialist doctors.^10^,^13^

The present study aimed to evaluate the satisfaction of dental interns toward their dental curriculum and education at colleges and universities in the central region of Saudi Arabia. To ensure curriculum development, continuous quality monitoring is mandatory because no curriculum remains fixed.^14^

Previous studies used different quantitative methods to evaluate the strength of the curriculum, including competency examinations, board examinations, oral examinations, students’ surveys, graduates’ surveys, instructors’ surveys, and patient satisfaction surveys.^7^ Surveys of graduates have an advantage since they are in a position to give important information about the strengths, weaknesses, and the importance of curriculum with its various modules.^7^,^15^ Surveys of graduates are essential to reveal their level of satisfaction with their profession, their practice patterns, and their learning behaviors.^7^,^16^ This is important since a dental curriculum aims to build confidence in students as well as to ensure that they acquire necessary skills. The survey on the clinical aspects revealed that most of the interns (48.4%) were highly satisfied with operative dentistry, while 17.4% were highly unsatisfied with orthodontic dentistry. This indicates that they were more satisfied with the common aspects of general dentistry and less satisfied with less frequent aspects.^17^-^21^

This finding concurs with the results reported in previous studies where dental students felt that they were inadequately prepared for practice in areas such as orthodontics. This is expected since orthodontic is a post-graduate subject.^1^,^22^,^23^ Regarding oral surgery and endodontics, Saudi students were mostly satisfied (35.8%) and highly satisfied (26.3%), which was in contrast to British students who reported deficient skills in oral surgery and endodontics.^1^ This difference might be due to the fact that Saudi students are exposed to more extraction cases than British students. It was also found that endodontic education has a lower priority in the United Kingdom dental curricula in comparison to Europe and the United States.^1^,^24^ Regarding the non-clinical aspects, students were highly unsatisfied (15.5%) with their research skills. Eslamipour et al. reported that more than half of their sample (dental students) were unsatisfied with their research skills.^25^ Another study found that only 17% of the dental students were satisfied with their research skills, which is very low in comparison to medical and pharmaceutical students.^26^

When comparison was made on gender, females had a higher non-clinical satisfaction score than males. This result differed from a finding reported in a previous study where no difference was found between the gender.^26^ That might be due to differences in the sample size since their sample included 62 participants. Moreover, in our study, gender played an important role since the students were studying at different campuses.

When we compared the interns in different colleges and universities in our city, there was a statistically significant difference in overall clinical satisfaction score (P ≤0.01), while no difference was found in the non-clinical satisfaction score. The highest satisfaction score was found among the interns that graduated from PNU (μ=76.884), followed by the interns from KSAUH (μ=73.140), and the interns from Majmaah University (μ=69.308). The lowest clinical satisfaction score was for KSU (μ=60.634). This might be due to differences in the curriculum; at PNS and KSAUH, the students start their comprehensive clinic during their third year while at KSU the students start specialty-based clinical training in their third and fourth years and they only have the comprehensive clinic in their fifth year. Majmaah University has the same curriculum as KSU, but it has a fewer number of students, which might affect the supervisor/student ratio and thus the students’ clinical satisfaction score.

### Limitations of the study

In the current study, the interns were asked about their satisfaction with different dental subjects. A study with more questions about the reason for their satisfaction or dissatisfaction, for example, the number of supervising faculty, the availability of materials and equipment, etc. The sample size is another limitation of this study. A future study with a larger sample is recommended. Including dental colleges and universities from different regions in Saudi Arabia is also recommended.

## CONCLUSION

In this study, the dental interns were more satisfied with the Operative Department and less satisfied with the Orthodontic Department. Females had a higher non-clinical satisfaction score than males. Dental students need a greater focus on and more exposure to research skills during their Dental School studies. To improve dental students’ level of clinical satisfaction, it is more important for them to have early exposure to comprehensive clinical training than specialty-based clinical training.

### Authors` Contribution:

**AHA, MAA, FSA and SFA** conceived, designed and did statistical analysis & editing of manuscript.

**AAA and IEA** did data collection and manuscript writing.

**AHA** did review and final approval of manuscript and is the author responsible for the accuracy or integrity of the work.
